# Quality of life measurement in cancer patients receiving palliative radiotherapy for symptomatic lung cancer: a literature review

**DOI:** 10.3747/co.v16i2.376

**Published:** 2009-03

**Authors:** N. Salvo, S. Hadi, J. Napolskikh, P. Goh, E. Sinclair, E. Chow

**Affiliations:** * Rapid Response Radiotherapy Program, Department of Radiation Oncology, Odette Cancer Centre, Sunnybrook Health Sciences Centre, University of Toronto, Toronto, ON.

**Keywords:** Lung cancer, quality of life, qol instrument, review, fact-L, eortc qlq-LC13, eortc qlq-C30

## Abstract

Approximately 27% of North American cancer deaths are attributable to cancer of the lung. Many lung cancers are found at an advanced stage, rendering the tumours inoperable and the patients palliative. Common symptoms associated with palliative lung cancer include cough, hemoptysis, and dyspnea, all of which can significantly debilitate and diminish quality of life (qol). In studies of the effects of cancer therapies, the frequent evaluative endpoints are survival and local control; however, it is imperative that clinical trials with palliative patients also have a qol focus when a cure is unattainable. We conducted a literature review to investigate the use of qol instrument tools in trials studying qol or symptom palliation of primary lung cancer or lung metastases through the use of radiotherapy. We identified forty-three studies: nineteen used a qol tool, and twenty-four examined symptom palliation without the use of a qol instrument. The European Organization for Research and Treatment of Cancer (eortc) qlq-C30 survey was the most commonly used qol questionnaire (in thirteen of twenty trials). Of those thirteen studies, eight also incorporated the lung-specific qol survey eortc qlq-LC13 (or the eortc qlq-LC17). A second lung-specific survey, the Functional Assessment of Cancer Therapy–Lung (fact-L) was used in only two of the twenty trials. In total, only ten of forty-three trials (23%) used a lung-specific qol tool, suggesting that qol was of low priority as an endpoint and that measures created for lung cancer patients are underused. We encourage investigators in future trials to include specific qol instruments such as the eortc qlq-LC13 or the fact-L for studies in palliative thoracic radiotherapy because those instruments provide a measure of qol specific to patients with lung cancer or lung metastases.

## 1. INTRODUCTION

Lung cancer is a rising epidemic and remains the leading cause of cancer death in both men and women in Canada^1^. In general, 500 Canadians are diagnosed with and 400 Canadians die of lung cancer every week^1^. Such high morbidity and mortality in patients with primary lung cancer emphasizes the need for palliative treatment intent.

Morbidity from lung cancer or lung metastases often presents as troublesome thoracic symptoms such as hemoptysis, cough, chest pain, and dyspnea. Palliative radiotherapy has been effective in ameliorating these symptoms 2–4 and improves or preserves the quality of life (qol) remaining in approximately one third of affected patients^5^.

In the past, clinical trials in patients with lung cancer have focused on traditional endpoints such as overall survival, disease-free survival, or local control 6. Given the relatively poor prognosis of patients with locally advanced lung cancer or lung metastases, the inclusion of qol as a primary endpoint of treatment becomes increasingly important. Quality of life encompasses the minimization of risks and maximization of benefits of a treatment, including physical and psychosocial effects on the well-being of patients^7^. Studying qol is particularly relevant in the field of palliative radiotherapy because of known treatment-related side effects and toxicities.

Few studies focus on qol and symptom palliation as primary endpoints. The purpose of the present review was to accurately assess the recent use of qol tools in trials that evaluated the efficacy of palliative radiotherapy in patients with lung cancer or lung metastases.

## 2. METHODS

### 2.1 Search Strategy

We conducted a literature review using the medline (Ovid) database for 1950 to February 2008. Key terms such as “lung cancer,” “lung neoplasms,” or “lung metastases” were combined with the terms “radiotherapy,” “radiation,” “external-beam irradiation,” or “palliative radiotherapy.” This search was then combined with “quality of life” or “qol” and also “symptom palliation.” Relevant articles and abstracts were reviewed, and references from those sources were also manually searched for additional relevant publications.

### 2.2 Inclusion Criteria

To be included in the present literature review, articles had to meet these criteria:

- Population: patients with a histologic, cytologic, or radiologic diagnosis of primary lung cancer or lung metastases
- Intervention: external beam radiotherapy or endobronchial brachytherapy in at least one study arm, with palliative intent
- Types of studies: randomized trials, prospective or retrospective cohort studies
- Endpoints: qol or symptom palliation as a primary or secondary endpoint or measured outcome

### 2.3 Exclusion Criteria

Articles were excluded if they met any of these criteria:

- Article type: individual case report or review article
- Language: publication in a language other than English
- Intervention: no evaluation, in at least one arm, of external beam irradiation to the thorax or endobronchial brachytherapy; or studies of interventions with curative intent
- Types of studies: focus on populations other than those with primary lung cancer or lung metastases
- Endpoints: use of the Karnofsky performance status (kps) or other similar prognostic tools, correlation of qol with cost–utility, or test of the reliability or validity of a qol instrument

### 2.4 Data Extraction

We extracted the following information from the studies:

- Primary and secondary outcomes
- Radiotherapy treatment details
- Type and number of qol, symptom palliation, and additional tools, if any, used
- Number of patients in each study arm
- Median age and male: female ratio of the patients enrolled in the study
- Median survival in each study arm

## 3. RESULTS

We identified a total of forty-three trials that evaluated, in at least one study arm, the use of palliative radiotherapy to the thorax, and that assessed qol or symptom palliation as a primary or secondary endpoint. Thirty studies (Table I) evaluated the treatment of patients with non-small-cell lung cancer (nsclc). Four studies (Table II) involved patients who were treated with endobronchial brachytherapy alone or in addition to external-beam radiation. Brachytherapy differs from external-beam radiation in that it is a more localized form of radiation that limits toxicity in healthy tissue to the immediate vicinity of the radiated region^5^. Another nine trials (Table III) evaluated the use of palliative radiotherapy in patients with lung cancer of a histologic type other than nsclc. The four identified studies that measured the difference in efficacy between endobronchial brachytherapy and external beam radiation 37–40 used both symptom palliation and qol scores as a primary outcome.

In twenty of the identified studies, symptom palliation was used as a primary outcome 8,10,11,13, 14,17,19,20,21,23,26,28–33,44,45,47. Ten trials used qol as a primary outcome 5,9,16,18,24,25,34,35,39,49, and six studies used both symptom palliation and qol together as a primary endpoint 22,27,37,38,40,48. Seven of the studies used neither symptom palliation nor qol as primary endpoints, but rather incorporated them as secondary outcomes 12,15,36,41–43,46. The four identified studies that measured the difference in efficacy between endobronchial brachytherapy and external beam radiation^37^–^40^ used both symptom palliation and qol scores as primary outcomes.

### 3.1 QOL and Symptom Palliation Tools Used

A total of 11 tools were used to assess either qol or palliation of lung cancer–related symptoms; the frequency of use of each tool is presented in Table IV. The most common qol tool used was the European Organization for Research and Treatment of Cancer (eortc) qlq-C30, a questionnaire that was created and validated to assess qol in individuals with any form of cancer. It has been translated into 81 languages and consists of 30 questions that encompass 5 functional scales: physical, role, cognitive, emotional, and social functioning^49^. The eortc qlq-C30 also incorporates 3 symptom scales: fatigue, pain, and nausea and vomiting. The remaining items on the questionnaire cover other symptom-related events that are often described by cancer patients, including dyspnea, diarrhea, and loss of appetite, among others^48^.

The eortc qlq-C30 was used in fourteen of the forty-three studies identified in the search (32%), eight of which also used the lung cancer supplement, eortc qlq-LC13. The eortc qlq-LC13 is the latest version of a lung cancer specific questionnaire that consists of questions concerning lung cancer symptoms and the side effects of conventional treatments used for lung cancer^49^. One trial used an older version of the lung-specific module, the eortc qlq-LC17, in addition to the general questionnaire^48^

The Functional Assessment of Cancer Therapy (fact) qol tools constituted a second group used in the identified studies. Both the general questionnaire (fact-G) and the lung-specific questionnaire (fact-L) were used. Like the eortc qlq-C30, the fact-G is a general questionnaire that was developed for pa­tients with any type of cancer. The fact-G covers 4 dimensions of qol: physical, social, emotional, and functional well-being^50^. The fact-L is similar to the eortc qlq-LC13 because it includes additional ques­tions that relate specifically to qol in patients with lung cancer. The fact-L was used in two studies, and the fact-G in one.

A third validated qol tool was used in one trial: the Spitzer qol Index. The Spitzer Index covers 5 dimensions of qol: activity, daily living, health, support of family and friends, and outlook^51^. It is not a lung cancer–specific questionnaire, however; and thus it does not incorporate questions directly related to the lung-cancer-specific patient population.

Study-designed questionnaires were the most prevalent tool used in the forty-three identified stud­ies. A study-specific method of determining qol was used in three trials, and nineteen trials attempted to evaluate symptom palliation using a study-designed questionnaire. Table V shows a breakdown of the proportion of studies using a validated qol or symptom palliation tool as compared with a study-designed tool. Study-designed instruments present a difficulty: drawing comparisons across studies is harder because the methods of measurement vary.

In five studies, a validated symptom palliation tool was used (the frequency of use can be seen in Table IV). The two general symptom tools used were the Hospital Anxiety and Depression Scale and the Rotterdam Symptom Checklist. The Rotterdam Symptom Checklist measures psychological and physical distress in cancer patients through the use of 38 items^52^. The Hospital Anxiety and Depression Scale is a tool used to measure anxiety and depression levels using 14 statements based on a patient’s experience over the preceding week^53^. One lung-specific symptom tool the Lung Cancer Symptom Scale was used. The Lung Cancer Symptom Scale is a tool designed to measure 6 lung-specific symptoms and their effects on symptomatic distress, functional burden, and global quality of life^54^,^55^.

**FIGURE 1:**
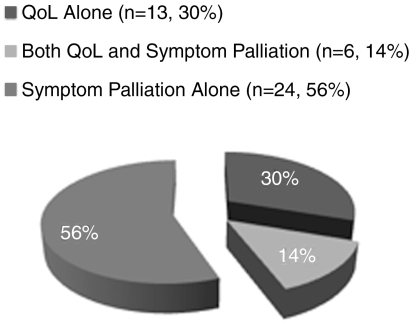
Questionnaire use in all identified studies.

Figure 1 outlines the overall picture of questionnaire use in the identified trials. Most of the trials (54%) measured symptom palliation alone; some measured both symptom palliation and qol (14%). The remaining trials measured qol only.

### 3.2 Performance Assessment

In forty studies (91%), the performance status of the subjects was measured in addition to qol or symptom palliation. Performance status was measured primarily as a prognostic factor (twenty of forty trials, 50%) or as part of the exclusion criteria (fourteen of forty trials, 35%). Only six studies used a performance scale as part of the assessment. The 3 most predominant performance status tools used were the World Health Organization performance status, the Eastern Cooperative Oncology Group scale, and the Karnofsky performance status (kps). Although performance scales are useful to determine the functional status of a patient, they are not adequate tools for measuring symptom palliation or qol.

## 4. DISCUSSION

In patients with terminal cancer, qol is a significant concept, and it is influencedby many factors, including symptoms, functional level, coping strategies, and support systems^51^. Common symptoms that influence a lung cancer patient’s qol include anxiety, depression, pain, fatigue, dyspnea, and cough^52^. Because lung cancer is the leading cause of cancer death in men and the second-leading cause in women globally^2^, it is important that qol is considered when caring for these patients.

Meaningful palliation refers to symptom relief and prolongation of good-quality survival in lung cancer patients^26^. When treating a patient with palliative intent, it is necessary to use tools that measure the intent of the treatment. For 86% of doctors from the United Kingdom, the United States, and Canada, the treatment of choice for patients with inoperable report of a lung cancer is palliative radiotherapy^33^. It is therefore important that, when considering the side effects of palliative radiotherapy as compared with the side effects of the lung cancer itself, trials investigating the use of palliative radiotherapy use a qol measure to determine the benefit of the treatment.

A total of twenty identified trials considering palliative radiotherapy for lung cancer included an evaluation of qol. Of these trials, eleven used a tool that was specific to patients with lung cancer; the remaining nine used general qol questionnaires for cancer patients or a study-designed questionnaire. In thirty-one identified studies, the level of symptom palliation, one aspect that contributes to a qol measure, was assessed. This finding suggests that more trials should use a validated lung-specific tool when evaluating the outcome of palliative thoracic radiotherapy. Use of a validated, lung-specific tool will allow for comparisons between trials and will also increase the internal validity of individual studies. Two recommended lung-specific validated tools that would be beneficial for the measurement of qol in trials evaluating palliative thoracic radiotherapy are the fact-L and the eortc qlq-LC13.

## Figures and Tables

**TABLE I:** Patients with inoperable non-small-cell lung cancer (nsclc) treated with palliative radiotherapy

Reference	Type	Study Purpose	Arms	Pts (*n*)	Median survival	qol	Assessment tools Performance	Other	Measures of qol (*n*)
Simpson *et al.*, 1985 8	rct (multicentre)	To evaluate 3 xrt schedules and determine the most efficient	A: 40 Gy split course in 4 weeks B: 30 Gy continuous for 2 weeks C: 40 Gy continuous for 4 weeks	316	A: 6.2 months B: 6.4 months C: 6.9 months	None	kps	Study designed: self-report either complete relief or relative relief by patient	0
Kaasa *et al.*, 1988 9	rct	qol of patients with radiation therapy and chemotherapy	A: Combination chemotherapy B: 42 Gy/15 fr	95	Not stated	Study designed: 29 variables; only psychosocial well-being and global qol reported	who	None	0
Teo *et al.*, 1988 10	rct	To compare a hypofractionated scheme with traditional fractionation	A: 45 Gy/18 fr B: 31.2 Gy/4 fr	291	Not stated	None	kps	Study designed: subjective responses in thoracic symptoms to changes	0
mrc Lung Cancer Working Party, 1991 11	Randomized prospective	To determine if a shorter treatment course of xrt provides equally good symptom palliation	A: 17 Gy/2 fr B: 30 Gy/10 fr	369	A: 179 days B: 177 days	None	who	Study designed: 4-point scale to rate symptoms	0
Regan *et al.*, 1991 12	Prospective	Correlate physician rating of xrt response to patient views of treatment	A: 30 Gy/10 fr B: 17 Gy/2 fr	40	30 Days	eortc qlq-C30	ecog	mrc physician questionnaire	1
mrc Lung Cancer Working Party, 1992 13	rct	Investigate whether a single fraction can provide palliation as good as that provided by 2 fractions	A: 17 Gy/2 fr B: 10 Gy/1 fr	233	A: 100 days B: 122 days	None	who	Study designed: daily dairy for first 6 months: 4-point scale to rate symptoms	0
Omand and Meredith, 1994 14	Prospective	To assess frequency of acute side effects of short-term xrt	A: 10 Gy/1 fr B: 17Gy/ 2 fr	61	Not stated	None	None	Study designed: percentage improvement in symptoms	0
Abratt *et al.*, 1995 15	Randomized prospective	To evaluate the dose–response effect on survival of patients with good performance status	A: 35 Gy/10 fr B: 45 Gy/15 fr	84	A: 8.5 months B: 8.5 months	None	who	Study designed: physician graded symptom improvements	0
Macbeth *et al.*, 1996^16^	Randomized (multicentre)	To compare palliative with more-intensive xrt with respect to survival and qol	A: 17 Gy/2 fr B: 39 Gy/13 fr	509	A: 7 months B: 9 months	None	who	hads, rscl mrc patient diary card	0
Ball *et al.*, 1997^17^	Prospective	To assess the effect of adding continuous-infusion fluorouracil to palliative xrt	A: 20 Gy/5 fr B: 20 Gy/5 fr with fluorouracil for 5 days	200	A: 6 months B: 6.8 months	Study-designed questionnaire	who	Study-designed questionnaire to detect symptom palliation	1
Gava *et al.*, 1997^18^	Prospective (multicentre)	To assess the indications for xrt, compliance with treatment plans, and qol	A: Radical range: 30Gy–70Gy B: Palliative range: <30 Gy to 70 Gy	A: 109 B: 73	Not stated	Study designed	kps	None	
Lutz *et al.*, 1997^19^	Retrospective	To measure symptom palliation in patients treated with xrt	30 Gy/10–12 fr	54	4 Months	None	swog performance status	lcss	1
Vyas *et al.*, 1998^20^	Retrospective	To evaluate response in patients receiving palliative xrt in 2 large fractions	17 Gy/2 fr	37	Not stated	None	Not stated	Study designed: patients asked to grade percentage improvement in symptoms	0
Donato *et al.*, 1999 21	Prospective	To examine the results obtained with a fractionated rt regimen	A: 20 Gy/5 fr (1 treatment) B: 40 Gy/10 (2 treatments)	52	Not stated	None	ecog, kps	Study designed: subjective patient assessment of symptoms	0
Langendijk *et al.*, 2000 5	Prospective	To see the association between prognostic factors and qol and the impact of symptoms on qol	A: Curative schedule: 70 Gy in 7 weeks B: Radical schedule: 60 Gy in 6 weeks C: Palliative schedule: 30 Gy in 4 weeks	262	A: 19.1 months B: 8.5 months C: 4.1 months	eortc-qlq-C30 eortc qlq-LC13	who	None	2
Langendijk *et al.*, 2000 22	Prospective	To investigate changes in symptoms and qol in patients receiving xrt	30 Gy/in 4 weeks	65	Not stated	eortc qlq-C30 eortc qlq-LC13	who	None	2
Nestle *et al.*, 2000 23	Randomized prospective	To see if there is a difference between palliative and more intensive treatment	A: 60 Gy/30 fr B: 32 Gy/20 fr	152	A: 8.3 months B: 8.4 months	None	kps	Study designed: mrc daily diary card	0
Schaafsma and Coy, 2000 24	Prospective	To estimate the effect of high-dose xrt on qol and computer qald gained	30 Gy/10 fr	54	266 Days	eortc qlq-C30	kps	None	1
Auchter *et al.*, 2001 25	Prospective	To evaluate qol of patients before, at completion, and after accelerated fractionation of xrt	57.6 Gy/36 fr over 15 days	30	13 Months	fact-L	ecog	None	1
BCentingoz *et al.* , 2001 26	Retrospective	To retrospectively evaluate the treatment effects of xrt	Median dose: 30 Gy/1–23 fr	115	30 Weeks	None	kps	Study designed: subjective palliation rates in one of three groups: near total response, improvement, or no response	0
Langendijk *et al.*, 2001 27	Prospective	To evaluate changes in qol and symptoms after xrt	60 Gy total dose	164	8.5 Months	eortc qlq-C30 eortc qlq-LC13	who	None	2
Bejzak *et al.*, 2002 28	rct (multicentre)	Comparison of 2 fractionation schedules on palliation of symptoms	A: 10 Gy/1 fr B: 20 Gy/5 fr	230	A: 4.2 months B: 6 months	eortc qlq-C30	ecog	lcss (1 item)	1
Falk et al., 2002 29	rct (multicentre)	To determine if patients should be given palliative xrt immediately or as needed for symptom relief	A: 17 Gy/2 fr B: 10 Gy/1 fr	230	A: 240 days B: 253 days	None	who	hads, rscl	0
Nihei *et al.*, 2002 30	Retrospective	To investigate the outcome of xrt for airway stenosis	30 Gy/10 fr	24	Responders: 192 days Non-responders: 43 days	None	None	Study designed: Patient subjective report of symptoms	0
Borthwick *et al.*, 2003 31	Prospective	To gain an understanding of fatigue in patients receiving xrt	A: Radical: 55 Gy/20 fr B: Palliative: 39 Gy/13 fr	53	Not stated	None	Not stated	Study designed: daily card with 9 questions relating to fatigue	0
Kramer *et al.*, 2005 32	rct (multicentre)	Compare various fractions of xrt on palliation of thoracic symptoms	A: 16 GY/2 fr B: 30 GY/10 fr	297	Not stated	None	ecog	rscl	0
Senkus–Konefka *et al.*, 2005 33	Randomized prospective	To compare two palliative xrt schedules	A: 20 Gy/5 fr B: 16 Gy/2 fr	100	A: 5.3 months B: 8.0 months	None	who	Study designed: patient-reported symptom relief on a 4-point scale	0
Sundstrøm *et al.*, 2005 34	Randomized prospective	To compare the course of symptoms and hr qol after immediate thoracic rt between symptomatic (Sym) and non-Sym (NSym) patients	17 Gy/2 fr 42 Gy/15 fr 50 Gy/25 fr	395	NSym: 11.8 months Sym: 6.0 months	eortc qlq-C30 eortc qlq-LC13	kps	None	2
Sundstrøm *et al.*, 2006 35	Randomized	To examine the predictive value of baseline hr qol data in patients receiving xrt in comparison with demographic, clinical, and treatment variables	A: 17 Gy/2 fr B: 42 Gy/15 fr C: 50 Gy/25 fr	301	A: 9.2 Months B: 7.5 Months C: 7.5 Months	eortc qlq-C30 eortc qlq-LC13	kps	None	2
Temel *et al* ., 2007 36	Prospective	To assess the feasibility of early palliative care in patients with newly diagnosed nsclc	Not stated	51	9.0 Months	fact-G fact-L	ecog	hads	2

Pts = patients; qol = quality of life; rct = randomized clinical trial; xrt = external-beam radiotherapy; kps = Karnofsky performance status; fr = fractions; who = World Health Organization; mrc = Medical Research Council; eortc = European Organization for Research and Treatment of Cancer; ecog = Eastern Cooperative Oncology Group; hads = Hospital Anxiety and Depression Scale; rscl = Rotterdam symptom checklist; swog = Southwest Oncology Group; lcss = Lung Cancer Symptom Scale; rt = radiotherapy; qald = quality-adjusted life-day; hr = health-related.

**TABLE II:** Patients with symptomatic lung cancer treated with endobronchial brachytherapy (ebb) as compared with external-beam radiotherapy (xrt) with or without ebb

Reference	Type	Study Purpose	Arms	Pts (*n*)	Median survival	qol	Assessment tools Performance	Other (*n*)	Measures of qol
Stout *et al.*, 2000 37	rct	To compare ebb and xrt for symptom palliation and the effect on functional status and qol of patients	A: 30 Gy/8 fr xrt B: 15 Gy/1 fr ebb	99	A: 287 days B: 250 days	None	who	Study designed: 4-point scoring system to monitor performance status and 9 key symptoms hads rscl modified for lung cancer	2
Langendijk *et al.*, 2001^38^	rct	To test that the addition of ebb to xrt provides higher levels of palliation of dyspnea and increases qol	A: xrt alone: 60 Gy/24 fr or 30 Gy/10 fr B: xrt (60 Gy/24 fr or 30 Gy/10 fr) plus ebb (15 Gy/2 fr)	95	A: 8.5 months B: 7.0 months	eortc qlq-C30 eortc qlq-LC13	who	None	2
Mallick *et al.*, 2006 39	Prospective	To test the hypothesis that palliative ebb treatment with or without xrt can reduce endobronchial symptoms for a prolonged period and also improve qol	A: 30 Gy/10 fr with ebb on days 6 and 13: 8Gy/1fr B: 30 Gy/10 fr with ebb on day 13: 10 Gy/1 fr C: ebb 15 Gy/1 fr	95	A: 10 months B: 10 months C: 10 months	eortc qlq-C30 eortc qlq-LC13	kps	None	2
Mallick *et al.*, 2007 40	Prospective	To compare the subjective and objective responses to 3 regimens for duration, qol outcomes, and complications	A: 30 Gy/10 fr with ebb on days 6 and 13: 8 Gy/1 fr B: 30 Gy/10 fr with ebb on day 13: 10 Gy/1 fr C: ebb 15 Gy/1 fr	45	Not stated	eortc qlq-C30 eortc qlq-LC13	kps	None	2

Pts = patients; qol = quality of life; rct = randomized clinical trial; fr = fractions; who = World Health Organization; hads = Hospital Anxiety and Depression Scale; rscl = Rotterdam symptom checklist; eortc = European Organization for Research and Treatment of Cancer; kps = Karnofsky performance status.

**TABLE III:** Patients with inoperable lung cancer [other than non-small-cell lung cancer (nsclc)] treated with palliative radiotherapy

Reference	Type	Study Purpose	Arms	Pts (*n*)	Median survival	qol	Assessment tools Performance	Other	Measures of qol (*n*)
Berry *et al.*, 1977^41^	Prospective	Compares xrt alone and with chemotherapy	A: 40 Gy/20 fr or 36 Gy/12 fr B: Single-agent continuous chemotherapy C: Intermittent quadruple chemotherapy	A: 48 B: 49 C: 51	125 Days	None	None	Study designed: physicians recorded changes in patient symptoms	0
Collins *et al.*, 1988 42	Prospective	To determine whether palliative rt should be given to a patient with inoperable carcinoma of the bronchus	Range: 18 Gy/5 fr–48 Gy/10 fr (split course)	96	38 Weeks	None	who	Study designed: symptom response questions	0
MRC Lung Cancer Working Party, 1989 43	rct (multicentre)	To compare two policies of treatment	A: Combination chemotherapy and xrt (40 Gy/15 fr) B: selective treatment: chemotherapy with or without xrt; treatment given as required to control symptoms	151	A: 32 weeks B: 16 weeks	None	who	Study designed: treatment reports and daily diary chart	0
Devereux *et al.*, 1997 44	Prospective	To assess the incidence and severity of the immediate side effects of palliative rt for bronchial carcinoma	Range: 8 Gy/1 fr–60 Gy/30 fr	118	Not stated	None	None	Study designed: questionnaire to determine occurrence of symptoms 24 hours post treatment	0
Rees *et al.*, 1997 45	Randomized prospective	To compare the symptomatic effects of two regimens of xrt	A: 17 Gy/2 fr B: 22.5 Gy/5fr	A: 111 B: 105	Not stated	None	who	Study designed: questionnaire to rate severity of symptoms	0
Ampil *et al.*, 2001^46^		To see the effects of palliative xrt on patients with synchronous bilateral lung cancers	Range: 5–58 Gy (mean dose: 35 Gy)	32	7 Months	None	swog	Study designed: subjective response	0
Erridge *et al.*, 2005 47	rct	To determine whether palliation of chest symptoms was the same in two fractionation schedules	A: 10 Gy/1 fr B: 30 Gy/10 fr	149	A: 28.3 Weeks B: 22.7 Weeks	Spitzer’s qol Index	who	hads ecog	1
Turner *et al.*, 2005 48	Prospective	To see if older people benefit from xrt treatment, both in control of symptoms and improvement in qol (nsclc, sclc, and unknown types)	A: “High dose”: (36/39 Gy in 12/13 fr) B: ‘‘low dose’’: (10 Gy in 1 fr, 17 Gy in 2 fr or 20 Gy in 5 fr)	Elderly (>75 years): 83 Younger (<65 years): 49	A: 9 months B: 7 months	eortc qlq-C30 eortc qlq-LC17	who, Barthel adl Scale	hads Concerns Checklist	2
Hicsönmez *et al.*, 2007 49		Evaluate efficacy of palliative xrt in terms of qol and how ecog correlates with eortc qlq-C30	Not stated	88	Not stated	eortc qlq-C30	ecog	None	1

Pts = patients; qol = quality of life; xrt = external-beam radiotherapy; fr = fractions; rt = radiotherapy; who = World Health Organization; mrc = Medical Research Council; rct = randomized clinical trial; swog = Southwest Oncology Group; hads = Hospital Anxiety and Depression Scale; ecog = Eastern Cooperative Oncology Group; sclc = small-cell lung cancer; eortc = European Organization for Research and Treatment of Cancer.

**TABLE IV:** Frequency of instruments used in clinical trials measuring quality of life (qol) in patients with locally advanced lung cancer or lung metastases

Instrument	Frequency
European Organization for Research and Treatment of Cancer (eortc)	
General cancer questionnaire (eortc qlq-C30)	13
Lung cancer questionnaire (eortc qlq-LC13)	7
Lung cancer questionnaire (eortc qlq-LC17)	1
Functional Assessment of Cancer Therapy (fact)	
General questionnaire (fact-G)	1
Lung questionnaire (fact-L)	2
Spitzer qlq Index	1
Hospital Anxiety and Depression Scale (hads)	5
Rotterdam Symptom Checklist (rscl)	4
Study-designed qlq questionnaire	3
Lung Cancer Symptom Scale (lcss)	2
Study-designed symptom palliation questionnaire	19

**TABLE V:** Use of validated or study-designed tools in forty-three studies

	Questionnaire type^a^			
	Symptom palliation		Quality of life	
	(*n*)	(%)	(*n*)	(%)
Validated	9	21	16	37
Study-designed	21	49	3	7
Total	30	70	19	44

a Six studies used both a qol and a symptom palliation tool.
